# Supplementary material for: Nudging accurate scientific communication
Source: PLoS One. 2023 Aug 31;18(8):e0290225. doi: 10.1371/journal.pone.0290225 (PMC10470889; doi:10.1371/journal.pone.0290225)
Supplement: S1 File — (DOCX) [file pone.0290225.s001.docx]

**Supplementary information**

*Nudging accurate scientific communication*

Allard, A., & Clavien, C.

Content

[**Appendix A: Materials for the Main vignettes** 2](#_Toc141115118)

[Motivation-Based Therapy [Experiment A = Low sample size, No RCT, Positive results. Experiment B = High sample size, RCT, Null results] 2](#_Toc141115119)

[Motivation-Based Therapy [Experiment A: Low sample size, RCT, Positive results. Experiment B: High sample size, RCT, Null results] 3](#_Toc141115120)

[Motivation-Based Therapy [Experiment: Low sample size, No RCT, Null results. Second experiment: High sample size, RCT, Positive results] 3](#_Toc141115121)

[Drug for hypertension 4](#_Toc141115122)

[Microfinance 4](#_Toc141115123)

[Critical Thinking Intervention 4](#_Toc141115124)

[Mindsets 5](#_Toc141115125)

[**Appendix B: Elicitation of plausibility of studied hypotheses 5**](#_Toc141115126)

[*Competing hypotheses* condition 5](#_Toc141115127)

[*Positive hypothesis only* condition 5](#_Toc141115128)

[**Appendix C: Exploratory items 6**](#_Toc141115129)

[Faith in intuition 6](#_Toc141115130)

[Scientific literacy 6](#_Toc141115131)

[**Appendix D: Robustness checks: Excluding the “new medical drug” condition 7**](#_Toc141115132)

[Study 1 7](#_Toc141115133)

[Study 2 8](#_Toc141115134)

#

# Appendix A: Materials for the Main vignettes

Note: we include on the associated OSF page the printed version of the Limesurvey questionnaires used for both experiments. We believe that the Limesurvey questionnaires are clear enough for most questions. However, due to the use of code for randomizing the vignettes, each vignette might be hard to read on the questionnaire (While the probes themselves are free of code). We thus reproduce here the five main vignettes.

We are showing a few possible variations for the first kind of vignette: Motivation-Based Therapy, to show how high sample size, presence of a control group, and positive results would sometimes come together in the same experiment, and would sometimes come apart.

For the four other vignette types, we show only one version of each vignette, but note that the following elements were randomized: university, sample size, use of a control group, and the existence of positive or null results. So for each vignette, we are only showing 1 of 16 possible versions.

Please also note that each vignette was preceded on the previous page by a quick description of each hypothesis and each intervention. These descriptions can be easily found on the limesurvey questionnaire.

## Motivation-Based Therapy [Experiment A = Low sample size, No RCT, Positive results. Experiment B = High sample size, RCT, Null results]

While conducting your research, you come across two experiments that have been conducted to test the efficiency of Motivation-Based Therapy.

Experiment A: Researchers at Yale university have recruited 40 participants from a local hospital because they suffered from depression. All the participants received Motivation-Based Therapy during three 1-hour sessions per week. After 5 weeks, the situation of 70% of participants had improved, as they had overall a better mood. 20% of participants showed no change, and the situation of 10% of participants deteriorated. The researchers concluded that the intervention was working.

Experiment B: Researchers at Princeton university have recruited 200 participants from a local clinic because they suffered from depression. Half of the participants received Motivation-Based Therapy during three 1-hour sessions per week. Half of the participants were put on a waiting list, with no treatment. After 5 weeks, around 50% of the participants had seen their condition improve in both the waiting-list group and the Motivation-Based Therapy group. These participants had overall a better mood. The researchers concluded that the intervention was not working.

## Motivation-Based Therapy [Experiment A: Low sample size, RCT, Positive results. Experiment B: High sample size, No RCT, Null results]

While conducting your research, you come across two experiments that have been conducted to test the efficiency of Motivation-Based Therapy.

Experiment A: Researchers at Princeton university have recruited 40 participants from a local clinic because they suffered from depression. Half of the participants received Motivation-Based Therapy during three 1-hour sessions per week. Half of the participants were put on a waiting list, with no treatment. After 5 weeks, the situation of 70% of participants had improved in the Motivation-Based Therapy group, as they had overall a better mood. However, in the waiting-list group, only 30% of participants had seen their condition improve. The researchers concluded that the intervention was working.

Experiment B: Researchers at Yale university have recruited 200 participants from a local hospital because they suffered from depression. All the participants received Motivation-Based Therapy during three 1-hour sessions per week. After 5 weeks, the situation of 30% of participants had improved, since they had overall a better mood. 40% of participants showed no change, and the situation of 30% of participants deteriorated. The researchers concluded that the intervention was not working.

## Motivation-Based Therapy [Experiment: Low sample size, No RCT, Null results. Second experiment: High sample size, RCT, Positive results]

While conducting your research, you come across two experiments that have been conducted to test the efficiency of Motivation-Based Therapy.

Experiment A: Researchers at Yale university have recruited 40 participants from a local hospital because they suffered from depression. All the participants received Motivation-Based Therapy during three 1-hour sessions per week. After 5 weeks, the situation of 30% of participants had improved, since they had overall a better mood. 40% of participants showed no change, and the situation of 30% of participants deteriorated. The researchers concluded that the intervention was not working.

Experiment B: Researchers at Princeton university have recruited 200 participants from a local clinic because they suffered from depression. Half of the participants received Motivation-Based Therapy during three 1-hour sessions per week. Half of the participants were put on a waiting list, with no treatment. After 5 weeks, the situation of 70% of participants had improved in the Motivation-Based Therapy group, as they had overall a better mood. However, in the waiting-list group, only 30% of participants had seen their condition improve. The researchers concluded that the intervention was working.

## Drug for hypertension

While conducting your research, you come across two experiments that have been conducted to test the efficiency of Xoliphenon.

Researchers from Yale university have recruited 40 participants from a local hospital because they had hypertension. All participants were given the new drug. After 5 days, the situation of 70% of participants had improved, since their blood pressure decreased; the situation of 20% of participants stayed the same, and the blood pressure of 10% of participants increased. The researchers concluded that the drug was working.

Experiment B: Researchers from Princeton university have recruited 200 participants from a local clinic because they had hypertension. Half of the participants were given the new drug and half a placebo. After 5 days, around 50% of the participants had seen their condition improve in both the control group and the placebo group, since their blood pressure decreased. The researchers concluded that the drug was not working.

## Microfinance

While conducting your research, you come across two experiments that have been conducted to test the impact of microfinance.

Experiment A: Researchers from Yale University partnered-up with an NGO to open a microlending center in 40 villages in rural India. They measured unemployment in these villages. They found that unemployment decreased after the microlending centers opened. The researchers concluded that the intervention was working.

Experiment B: Researchers from Princeton University partnered-up with an NGO to open a microlending center in 200 villages in rural India. They had an initial list of 200 villages; they randomly selected half of the villages to open microlending centers there, and half to be control villages, without microlending centers. Researchers measured unemployment in all villages. They found no difference in unemployment between the villages where microlending centers opened and the villages where no microlending center opened. The researchers concluded that the intervention was not working.

## Critical Thinking Intervention

While conducting your research, you come across two experiments that have been conducted to test the impact of Critical Thinking programs.

Experiment A: Researchers from Yale University have recruited 40 undergraduate students and submitted them to a Critical Thinking program. They measured how many fake news participants shared on Twitter. They found that students shared less fake news after the intervention compared to before the intervention. The researchers concluded that the intervention was working.

Experiment B: Researchers from Princeton University have recruited 200 undergraduate students in a Critical Thinking program. Half of them participated in a fake news training program, and half of them were kept as a control group and received no training. They measured how many fake news participants shared on Twitter. They found that participants were equally likely to share fake news in both the control group and the training group. The researchers concluded that the intervention was not working.

## Mindsets

While conducting your research, you come across two experiments that have been conducted to test the impact of mindsets.

Experiment A: Researchers from Yale university have recruited 40 participants from a local school. All students were assigned to a growth mindset intervention. Students read materials about the plasticity of the brain and how their intelligence changes over time. Six months later, researchers collected their grades. They found that students had improved their grades compared to the beginning of the term. The researchers concluded that the intervention was working.

Experiment B: Researchers from Princeton university have recruited 200 participants from a local school. Half of the students were assigned to a growth mindset intervention, and half of the students were left as a control group, without intervention. Students in the intervention group read materials about the plasticity of the brain and how their intelligence changes over time. Six months later, researchers collected their grades. They found that students assigned to the growth mindset intervention had no better grades than students in the control group. The researchers concluded that the intervention was not working.

# Appendix B: Elicitation of plausibility of studied hypotheses

## *Competing hypotheses* condition

What is your intuition regarding these hypotheses?

- Hypothesis A is definitely true.
- Hypothesis A is probably true.
- Both hypotheses are equally likely to be true.
- Hypothesis B is probably true.
- Hypothesis B is definitely true.

## *Positive hypothesis only* condition

What is your intuition regarding this hypothesis?

- This hypothesis is definitely true.
- This hypothesis is probably true.
- This hypothesis is equally likely to be false or true.
- This hypothesis is probably false.
- This hypothesis is definitely false.

# Appendix C: Exploratory items

For both scales, participants had the choice between the following five options:

- Disagree strongly
- Disagree moderately
- Neither agree nor disagree
- Agree moderately
- Agree strongly

## Faith in intuition

I trust my initial feelings about the facts.

I can usually feel when a claim is true or false even if I can’t explain how I know.

I trust the facts, not my instinct, to tell me what is true. (Reversed)

I need to be able to justify my beliefs with evidence. (Reversed)

## Scientific literacy

If I take medication, and I feel better in a few days, that's very strong evidence that the medication is working. (Reversed)

To measure the impact of an intervention, it is essential to compare two groups: one with, and one without, the intervention.

If my neighbour takes a new medication for the flu, and she recovers after a few days, that’s strong evidence that the medication is working. (Reversed)

#

# Appendix D: Robustness checks: Excluding the “new medical drug” condition

## Study 1

| ***Table S1:*** *Predicting preference for first experiment based on methodological features and positive results.* | | | | | |
| --- | --- | --- | --- | --- | --- |
| Predictor | $b$ | 95% CI | $t$ | $df$ | $p$ |
| Intercept | 2.32 | [2.17, 2.47] | 30.64 | 580 | < .001 |
| RCT | 0.66 | [0.51, 0.81] | 8.71 | 580 | < .001 |
| High Sample Size | 0.35 | [0.20, 0.50] | 4.64 | 580 | < .001 |
| Positive Results | 0.36 | [0.21, 0.51] | 4.75 | 580 | < .001 |

| ***Table S2:*** *Predicting preference for first experiment based on methodological features, positive results, and nudges.* | | | | | |
| --- | --- | --- | --- | --- | --- |
| Predictor | $b$ | 95% CI | $t$ | $df$ | $p$ |
| Intercept | 2.19 | [1.85, 2.52] | 12.74 | 564 | < .001 |
| Positive Results | 0.19 | [-0.17, 0.55] | 1.05 | 564 | .294 |
| Positive Hypothesis Only | 0.21 | [-0.10, 0.51] | 1.33 | 564 | .183 |
| Social Role: Impact | 0.12 | [-0.31, 0.56] | 0.56 | 564 | .578 |
| Social Role: Interest | 0.02 | [-0.40, 0.45] | 0.11 | 564 | .913 |
| Social Role: Accurate | -0.06 | [-0.49, 0.37] | -0.28 | 564 | .777 |
| RCT | 0.76 | [0.40, 1.11] | 4.18 | 564 | < .001 |
| High Sample Size | 0.36 | [0.01, 0.72] | 2.00 | 564 | .046 |
| Positive Results $\times$ Positive Hypothesis Only | 0.01 | [-0.30, 0.31] | 0.05 | 564 | .964 |
| Positive Results $\times$ Social Role: Impact | 0.04 | [-0.40, 0.48] | 0.18 | 564 | .861 |
| Positive Results $\times$ Social Role: Interest | 0.20 | [-0.23, 0.64] | 0.93 | 564 | .351 |
| Positive Results $\times$ Social Role: Accurate | 0.33 | [-0.10, 0.76] | 1.51 | 564 | .132 |
| Positive Hypothesis Only $\times$ RCT | 0.00 | [-0.31, 0.30] | -0.02 | 564 | .985 |
| Social Role: Impact $\times$ RCT | -0.03 | [-0.48, 0.41] | -0.14 | 564 | .888 |
| Social Role: Interest $\times$ RCT | -0.30 | [-0.73, 0.13] | -1.36 | 564 | .175 |
| Social Role: Accurate $\times$ RCT | -0.01 | [-0.44, 0.42] | -0.05 | 564 | .960 |
| Positive Hypothesis Only $\times$ High Sample Size | -0.18 | [-0.48, 0.12] | -1.18 | 564 | .239 |
| Social Role: Impact $\times$ High Sample Size | 0.05 | [-0.40, 0.50] | 0.21 | 564 | .835 |
| Social Role: Interest $\times$ High Sample Size | 0.26 | [-0.17, 0.69] | 1.19 | 564 | .234 |
| Social Role: Accurate $\times$ High Sample Size | 0.08 | [-0.35, 0.51] | 0.35 | 564 | .723 |

| ***Table S3:*** *Predicting preference for first experiment based on methodological features, positive results, and agreement with the hypothesis.* | | | | | |
| --- | --- | --- | --- | --- | --- |
| Predictor | $b$ | 95% CI | $t$ | $df$ | $p$ |
| Intercept | 2.43 | [2.22, 2.63] | 23.34 | 538 | < .001 |
| Positive Results | 0.05 | [-0.18, 0.29] | 0.45 | 538 | .653 |
| Intuition centered | -0.10 | [-0.26, 0.05] | -1.27 | 538 | .204 |
| RCT | 0.63 | [0.47, 0.78] | 8.04 | 538 | < .001 |
| High Sample Size | 0.34 | [0.19, 0.50] | 4.38 | 538 | < .001 |
| Positive Results $\times$ Intuition centered | 0.36 | [0.14, 0.57] | 3.29 | 538 | .001 |

## Study 2

| ***Table S4:*** *Predicting preference for first experiment based on methodological features and positive results.* | | | | | |
| --- | --- | --- | --- | --- | --- |
| Predictor | $b$ | 95% CI | $t$ | $df$ | $p$ |
| Intercept | 2.45 | [2.34, 2.56] | 44.04 | 933 | < .001 |
| RCT | 0.29 | [0.19, 0.40] | 5.43 | 933 | < .001 |
| High Sample Size | 0.37 | [0.26, 0.48] | 6.86 | 933 | < .001 |
| Positive Results | 0.47 | [0.37, 0.58] | 8.75 | 933 | < .001 |

| ***Table S5:*** *Predicting preference for first experiment based on methodological features, positive results, and nudges.* | | | | | |
| --- | --- | --- | --- | --- | --- |
| Predictor | $b$ | 95% CI | $t$ | $df$ | $p$ |
| Intercept | 2.58 | [2.37, 2.79] | 24.16 | 921 | < .001 |
| Positive Results | 0.29 | [0.08, 0.50] | 2.75 | 921 | .006 |
| Positive Hypothesis Only | 0.03 | [-0.18, 0.25] | 0.31 | 921 | .755 |
| Pressure towards Quality | -0.10 | [-0.37, 0.16] | -0.78 | 921 | .438 |
| Pressure towards Sales | -0.36 | [-0.62, -0.10] | -2.67 | 921 | .008 |
| RCT | 0.26 | [0.05, 0.47] | 2.45 | 921 | .015 |
| High Sample Size | 0.33 | [0.12, 0.54] | 3.10 | 921 | .002 |
| Positive Results $\times$ Positive Hypothesis Only | 0.12 | [-0.09, 0.33] | 1.15 | 921 | .249 |
| Positive Results $\times$ Pressure towards Quality | -0.11 | [-0.37, 0.14] | -0.87 | 921 | .387 |
| Positive Results $\times$ Pressure towards Sales | 0.47 | [0.21, 0.72] | 3.60 | 921 | < .001 |
| Positive Hypothesis Only $\times$ RCT | -0.02 | [-0.24, 0.19] | -0.23 | 921 | .818 |
| Pressure towards Quality $\times$ RCT | 0.08 | [-0.18, 0.34] | 0.61 | 921 | .544 |
| Pressure towards Sales $\times$ RCT | 0.08 | [-0.17, 0.34] | 0.64 | 921 | .525 |
| Positive Hypothesis Only $\times$ High Sample Size | -0.09 | [-0.30, 0.12] | -0.80 | 921 | .425 |
| Pressure towards Quality $\times$ High Sample Size | 0.11 | [-0.15, 0.37] | 0.84 | 921 | .401 |
| Pressure towards Sales $\times$ High Sample Size | 0.16 | [-0.09, 0.42] | 1.26 | 921 | .209 |

| ***Table S6:*** *Predicting preference for first experiment based on methodological features, positive results, and agreement with the hypothesis.* | | | | | |
| --- | --- | --- | --- | --- | --- |
| Predictor | $b$ | 95% CI | $t$ | $df$ | $p$ |
| Intercept | 2.52 | [2.39, 2.64] | 38.46 | 858 | < .001 |
| Positive Results | 0.34 | [0.20, 0.48] | 4.83 | 858 | < .001 |
| Belief in the hypothesis | -0.11 | [-0.21, -0.01] | -2.14 | 858 | .033 |
| RCT | 0.28 | [0.17, 0.39] | 5.10 | 858 | < .001 |
| High Sample Size | 0.35 | [0.24, 0.46] | 6.35 | 858 | < .001 |
| Positive Results $\times$ Belief in the hypothesis | 0.30 | [0.15, 0.46] | 3.90 | 858 | < .001 |
